# Editorial: Exploration of the role of heme proteins in biology with experimental and computational methods

**DOI:** 10.3389/fchem.2026.1810989

**Published:** 2026-02-25

**Authors:** Dragan M. Popović, Sergey A. Siletsky, Chunmao He, M. R. Gunner

**Affiliations:** 1 Institute of Chemistry, Technology, and Metallurgy, National Institute of the Republic of Serbia, University of Belgrade, Belgrade, Serbia; 2 Belozersky Institute of Physical-Chemical Biology, Lomonosov Moscow State University, Moscow, Russia; 3 School of Chemistry and Chemical Engineering, South China University of Technology, Guangzhou, China; 4 Physics Department, City College of New York, CUNY, New York, NY, United States

**Keywords:** bioenergetics, CYP450 enzymes, cytochrome nanowires, cytochrome oxidases, heme, molecular simulation methods, protein dysfunction, reaction mechanisms

Heme proteins are a diverse group of metalloproteins that use an iron–porphyrin cofactor to perform vital biological functions, including oxygen transport and storage, electron transfer (ET), catalysis, detoxification, sensing of O_2_, NO and CO and signal transduction (Shimizu et al., 2015), and proton pumping and energy transduction. Their functional diversity arises from precise control of the heme iron’s oxidation and spin state, coordination environment, and redox properties as dictated by the surrounding protein matrix (Popović and Đorđević, 2022; Zheng and Gunner, 2009). This Research Topic explores the structural and functional diversity of heme proteins, emphasizing key experimental and computational methods for understanding their catalytic properties (Popović, 2022), physicochemical characteristics (Guberman-Pfeffer et al., 2024), and reaction mechanisms (Cai et al., 2020; Medvedev et al., 2008; Siletsky, 2023).

Studying how these biomolecules function can help design more efficient drugs or inhibitors and develop more effective treatments for disorders related to the clinically relevant mutations and dysfunction of heme proteins (Lee et al., 2024; Zhai et al., 2023). Synthetic bioinspired heme proteins are valuable model systems in structural/functional studies (Hardy and Curnow, 2024) and have potential for various biomedical, biotechnological, and biosensing applications (Chen et al., 2023; Dogutan and Nocera, 2019; Gondim et al., 2022; Korendovych and DeGrado, 2020).

The molecular mechanism of proton pumping in cytochrome *c* oxidases (CcOs) has been the subject of long-standing debate. The present work by Shimada et al. analyzes recent high-resolution structures and highlights the conservation of the proton-conducting H-channel across different CcO families. Their model offers another perspective on proton unidirectionality and how the enzyme prevents backflow.

Despite discussions regarding the role of the D- vs. the H-channel in proton transport, several questions remain unresolved. The lack of a conventional D-channel in the *ba*_3_ systems raises concerns about the applicability of the same mechanism across all three enzyme families (A, B, and C). Variations in proposed mechanisms within the A family (*aa*_3_) further complicate the issue. The article encourages a more thorough examination of the H-channel role in proton pumping and revitalizes interest in the subject, reminding readers that specific uncertainties about the proton-pumping mechanism persist.

Tabari and Hochbaum review provides a comprehensive overview of extracellular ET mechanisms in *Geobacter sulfurreducens*, with a particular focus on the roles of multiheme *c*-type cytochromes (MHCs) and the molecular pathways enabling trans-envelope electron transport. The paper integrates structural, mechanistic, and functional insights related to MHC nanowires (OmcS, OmcE, and OmcZ) and their evolving role in long-range electron transport. It outlines current knowledge gaps and emerging research directions in microbial bioelectrochemical systems and their relevance to biogeochemical cycling, bioenergy, and biotechnology.

In their study, Guberman-Pfeffer and Herron examine the electron conduction properties of cytochrome nanowires (CNs) used by mineral-respiring microorganisms. The theoretical and functional aspects of electron transport are analyzed using advanced computational methods to assess the physiological efficacy of these filaments as electrical conduits for extracellular ET. The findings reveal that CNs exhibit weak electronic coupling, strong redox anti-cooperativity, and sub-optimal conductivities. Although individual nanowires carry limited current, hundreds of filaments are sufficient for cellular respiration. This work challenges some interpretations of CN conductivity and provides a more physically realistic framework for understanding ET in multiheme cytochromes. It critically contributes to rationalizing the diverse results reported in the literature.

The cytochrome P450 (CYP450) family of heme-containing enzymes is primarily found in the liver and plays crucial roles in metabolizing endogenous and exogenous compounds, xenobiotics, drugs, and alcohol. Zhu et al. conducted a detailed biochemical study to investigate the roles of different CYP450 enzymes in alcoholic fatty liver disease (AFLD) using a sophisticated rat model that simulates the influence of human alcohol consumption and dietary variations. The specific effects of long-term ethanol exposure on the expression profiles of CYP450 enzymes and ATP-dependent membrane efflux pump (P-glycoprotein) were identified. These findings may guide future therapeutic strategies targeting CYP450 enzymes to prevent and treat AFLD.

The short review (Sil and Chakraborti) highlights the roles of hemoglobin and myoglobin in oxidative stress, detailing how they release heme and free iron under stress conditions, which can lead to oxidative damage via the Fenton reaction. This process is linked to various diseases, including metabolic syndrome, diabetes, cardiovascular, neurodegenerative and kidney diseases. The text emphasizes the importance of understanding iron metabolism, as it plays a dual role as a vital cofactor and a potential biohazard. Several studies indicate that heme iron is involved in disease progression, suggesting that a better understanding of the mechanisms underlying heme proteins and oxidative stress could help develop future therapies.

Recent studies indicate that iron ions bound to proteins and cofactors involved in iron metabolism might play a critical role in the development of Alzheimer’s disease (AD). Kaviyarasu et al. adopted a comprehensive approach to explore potential anti-Alzheimer compounds from *Cardiospermum halicacabum* leaves using high-throughput virtual screening, MD simulations, and gas chromatography-mass spectrometry. To optimize the extraction process, they employed microwave-assisted extraction and identified 40 phytoconstituents from the ethanolic extract; 37 were newly reported. These compounds demonstrated drug-like pharmacological properties, stability in MD simulations, and favorable electronic properties in DFT analysis. The results suggest that these phytocompounds could contribute to the development of novel natural therapeutic agents for AD.

The article (Ed-Dahmani et al.) presents a thorough phytochemical analysis of the extract from *Ferula communis* leaves, a medicinal plant widely used in traditional medicine, particularly for its antioxidant properties and high levels of polyphenols and flavonoids. The study aims to investigate antioxidant capacity (DPPH and FRAP methods), fully characterize the extract using UHPLC-MS/MS methods, evaluate toxicity through *in vivo* and *in silico* analyses, and predict the pharmacokinetics (ADMET) of the isolated compounds, also indicating their good absorption potentials.

The study by da Silva et al. demonstrates that when iron or heme availability is restricted in *Bacteroides thetaiotaomicron*, the bacterium adapts its metal-uptake mechanisms, leading to a notable increase in manganese accumulation. This adaptive response demonstrates how iron and manganese can substitute for each other in bacterial metabolism, particularly during oxidative stress or nutrient scarcity. Metallomic analyses by HPLC and high-resolution ICP-MS provided compelling evidence that bacteria can maintain essential cellular functions by utilizing manganese in the absence of iron. This work contributes to understanding microbial metal homeostasis and its evolutionary implications for gut microbiota adaptation.

Andrographolide (AGP) can reduce inflammatory responses in skin diseases by hindering the release of inflammatory factors such as TNF-α and IL-6. In a computational study, Lin et al. examined the interactions between AGP and *Cutibacterium acnes*, primarily using DFT methods to analyze its inhibitory bioactivity. The authors have combined molecular docking and MD simulation results with quantum-mechanical methods to examine this topic.

Heidinger et al. combined EPR experiments and computer modeling to clarify the mechanism of AhbD heme synthase that catalyzes the final step of the heme biosynthetic pathway in archaea and sulfate-reducing bacteria. AhbD uses radical chemistry to decarboxylate propionate side chains of iron-coproporphyrin III into vinyl groups of heme *b*. The DFT calculated EPR spectra helped elucidate the structures of the porphyrinyl radicals and led to key conclusions about AhbD’s oxidative decarboxylation mechanism. Specifically, the hydrogen-atom abstraction site was identified at the β-position of the propionate side chain, and the central iron ion and nearby ligand were shown to participate in ET to the iron-sulfur cluster in AhbD.

Covering all advancements in hemoproteins is a challenging task. The purpose of this Research Topic is to highlight current directions in key areas of heme protein chemistry and showcase contemporary applications of both experimental and computational methods. The selected papers illustrate the dynamic research activity in this field. Some contributions build upon ongoing debates, while others introduce novel perspectives that may challenge established interpretations. It is essential to recognize that the diversity of viewpoints from different research groups can stimulate constructive discussion, ultimately deepening our understanding of these remarkable protein systems and helping the field move forward.

Upcoming research on heme proteins will span from fundamental biochemistry to translational applications: from heme’s role in signaling and diseases, through engineering microbes for the production of heme and heme derivatives, to the advanced design of heme-based biocatalysts, new medicines, and materials. This underscores heme’s central role in both life processes and emerging technologies. Additionally, new experimental techniques, computational methodologies, and advances in artificial intelligence, machine learning, and supercomputer capabilities are expected to propel further progress in this field.
